# Specific steps in the operation determine resident speed: Experience with a live tissue simulation model of laparoscopic nephrectomy

**DOI:** 10.3389/fsurg.2022.997324

**Published:** 2022-10-20

**Authors:** Jackson Eber, Andrew C. Peterson

**Affiliations:** ^1^ University of Tennessee Health Science Center College of Medicine, Memphis, TN, United States; ^2^ Duke University Medical Center, Department of Surgery, Division of Urology, Durham, NC, United States

**Keywords:** LPNx: laparoscopic partial nephrectomy, LRNx: laparoscopic radical nephrectomy education, laparoscopy, training, urology, nephrectomy

## Abstract

**Introduction:**

It is increasingly important to identify and eliminate inefficiencies in resident education. We hypothesize that slower performance of specific operative steps in laparoscopic nephrectomy accounts for much of the slower operative speed observed in junior residents vs. their senior colleagues. Therefore, we sought to evaluate the by-step time-differential between experienced senior residents and their junior colleagues in a live-tissue simulation.

**Materials and methods:**

Residents participated in our swine model surgical simulation of laparoscopic radical and partial nephrectomy (LRNx and LPNx). PGY5 and 6 residents were considered senior; junior residents were PGY3 and 4. We defined discrete surgical steps. Residents' post-graduate training levels were tracked and time-to-completion of each operative step was recorded.

**Results:**

Seven live-tissue simulations sessions took place, with 12 residents conducting 22 operations (12 LRNx, 10 LPNx). On average, each resident operated in 2 simulation sessions (range 1–4). The average time required by senior residents for LPNx was 152 min; junior residents required 173 min (*p* = 0.35). When considering the operative steps, juniors required nearly twice as much time to achieve hilar control (42 min vs. 23 min, *p* = 0.03). Significant differences in performance time were not seen in the other steps.

**Discussion:**

The performance differential between senior and junior residents conducting nephrectomies was most evident during hilar dissection. Our study suggests that specific efforts should be focused on teaching junior residents the skills required for this step early in their training.

## Introduction

To survive the mounting pressure to train residents in a manner that is safe, effective and cost-efficient, urology residencies must move beyond the traditional surgical training model rooted in the Halstedian principles of the master-apprentice relationship (1). Work hour restrictions, the increasing demand for sub specialization, and the rapid deployment of new surgical methods and devices have rendered obsolete the notion that trainees will gain the necessary range and mastery of operative skills by simple observation and repetition (2). Furthermore, while surgical skills comprise the critical mass of a surgical resident's educational curriculum, the operative theatre is an expensive and potentially risky classroom. As a result, surgical proficiency must now be acquired in less time and, given the public's increasing attention to the performance of physicians, under greater scrutiny (3). To remain successful, graduate medical training programs must explore structured methods to teach and evaluate procedural and operative skills.

Modern surgical simulation allows trainees to master basic skills, simulate key intraoperative maneuvers, exercise critical thinking, and even treat emergency situations before entering the operating room (4–6). Moreover, training on simulators offers a unique opportunity to provide targeted feedback to the trainee based on objective measurements and, in aggregate, may increase our understanding of surgical education. Knowledge of how surgeons best acquire new skills can be applied to more efficiently training the next generation of residents, as well as to determining how to best integrate new technologies into clinical practice (7, 8).

Given increasing constraints on resident operative experience and buoying interest in simulated surgical training, we began a series of live-tissue simulation laparoscopic partial and radical nephrectomy (LPNx and LRNx, respectively) at our institutions. We hypothesized that live-tissue simulation offers a unique opportunity to isolate specific operative steps that especially challenge residents and sought to identify those steps in which the time-to-completion differential between junior and senior residents was greatest. Once these steps are identified, we feel that specific attention may be directed toward them, paying valuable future dividends in overall improvement in resident surgical skill acquisition.

## Materials and methods

### Settings and participants

We conducted an institutional animal care and use committee (IACUC) approved live-tissue surgical simulation training program at our institution. This 2-week progressive surgical simulation curriculum included didactic instruction, inanimate simulation, and live-tissue swine models of laparoscopic surgery. Swine were selected for their high fidelity to human renal anatomy, particularly in the region of the renal hilum. Participants were recruited from the six-year urology residency programs at our institutions. Trainees were encouraged, but not required, to participate regardless of level of training, perceived operative skills, or previous experience with laparoscopic and/or simulated surgery.

### Intervention

Multiple sessions were conducted; however, session structure remained stable over time. On day one, residents were given introductory didactic lectures, as well as assignments for independent reading. Didactic instruction focused on the guiding principles of live-tissue simulation, namely conscientious and ethical use of animals for surgical simulation modeling. Participants also viewed a presentation regarding laparoscopic surgical techniques. Independent reading material was selected to reinforce these concepts.

The following week, on day 8, residents once again gathered for a mentored skills lab, using inanimate box trainer surgical simulators. Specific skills applicable to nephrectomy were taught, and residents were given access to the box trainer for optional independent practice during days 9–13. On day 14, all participants took part in the live-tissue simulation. Once the didactic and inanimate training sessions were complete, the participants transitioned to the live-tissue simulation portion of the training. Residents were informed which surgical procedure(s) they would perform as the “primary surgeon.” When not acting as the “primary surgeon,” residents were instructed to work as the “assisting surgeon” under the guidance of the primary surgeon. Typically, laparoscopic radical nephrectomies (LRNx) were assigned to junior residents and laparoscopic partial nephrectomies (LPNx) were assigned to senior residents, however, some overlap did occur based on participant availability and a surplus of swine during one testing session.

One senior and one junior resident then were randomly paired and assigned to a single anesthetized swine. Each pair was monitored by a faculty urologist, who was instructed to time the various operative steps, probe the trainee's knowledge, and evaluate the trainee's operative plan, surgical skills and management of the operative team utilizing surgical procedure score sheets specific to each operation. Faculty were permitted to provide verbal guidance when it was necessary to move trainees beyond an impasse, however they were forbidden from directly participating in the operation in any way.

### Outcomes

Each operative procedure was timed, and the duration required for each operative step by each resident was recorded. Residents were aware that their performance was being timed, and no *a priori* goal or maximum time was set. For those completing a radical nephrectomy, the steps of the operation were delineated as follows: creation of pneumoperitoneum, trocar placement, mobilization of adjacent structures, isolation of the ureter and gonadal vein, obtainment of hilar control, and removal of the kidney. The initial steps, through obtainment of hilar control, remained the same for those completing a partial and total nephrectomy; however, in the partial nephrectomy operation, the additional steps of lesion removal and achievement of hemostasis were also evaluated. Hilar control included dissection and isolation of the renal artery and vein, obtaining control of the artery and vein with vessel loops, application of vascular clamps on each (for partial nephrectomy) and endoscopic stapling of the vessel (in complete nephrectomy groups). The simulated lesion in LPNx was a standardized 2 × 2 cm area of the upper pole that was scored with electrocautery by the observing attending.

### Analysis

**Figure 1:**
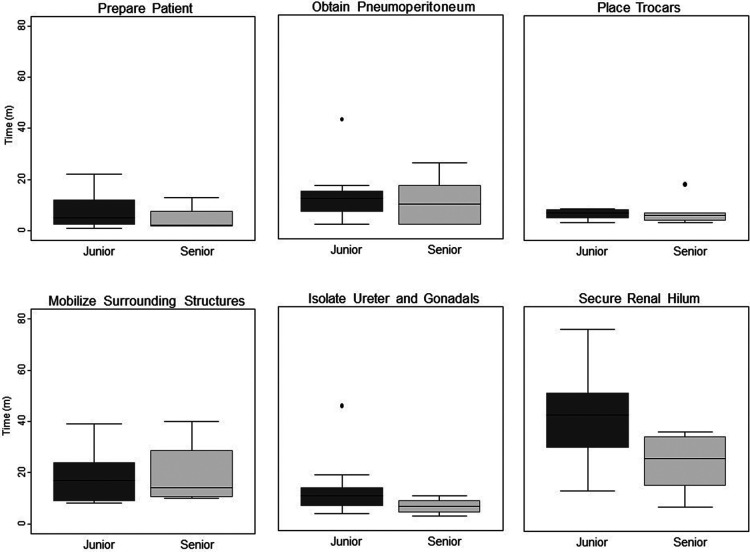
Time differential between junior vs. senior residents completing key steps in simulated laparoscopic radical and partial nephrectomy.

Once all groups had completed the simulation activities, times required for completion of each step of each LRNx and LPNx were compiled. Residents in their fifth or sixth year of training were considered senior residents; all others were considered junior residents. Appropriate parametric and non-parametric tests were employed, based on data characteristics. Comparisons of time-requirements between groups were conducted using Student's t-test and recorded in Figure 1. A *p*-value below 0.05 was considered statistically significant and all alphas were two-tailed. Statistical analysis was performed using Stata 15 (Stata Corp, College Station, TX).

### Oversight

The IACUC approved this study. Given that this was a non-risk intervention in medical education, the Institutional Review Board (IRB) deemed no official IRB oversight to be required. In lieu of official IRB oversight, the principles of the Declaration of Helsinki were followed.

## Results

Seven live-tissue simulations took place, with 12 urological surgical trainees conducting 22 operations in total. Of these, 12 were LRNx and 10 were LPNx. On average, each trainee operated in 2 simulations (range 1–4). In total, 15 cases were performed by junior residents (of these, two were post graduate year (PGY) 2, six PGY 3, and seven PGY 4) and 7 cases were performed by senior residents (two were PGY 5 and five PGY 6). LRNx was considered to be simpler operation, and was therefore geared toward junior residents, who performed 91.6% (11/12) of these procedures. LPNx was considered to be a more complex operation, and the majority of these cases (6/10, 60.0%) were performed by senior residents. None of the junior residents had performed prior LRNx or LPNx in humans as the primary surgeon, whereas all of the senior residents had performed these procedures previously in the clinical setting.

The junior residents required an average of 125 min to complete the LRNx. Given that only a single senior resident completed LRNx, we did not compare overall procedure time between junior and senior residents for this operation. The average time required by senior residents to complete an LPNx was 152 min as compared to 173 min for junior residents, though this difference was not statistically significant (*p* = 0.35).

When key components of the operations were compared between junior and senior residents, the junior residents required nearly twice as much time to achieve hilar control, taking 42 min, vs. 23 min for senior residents (*p* = 0.03). Senior residents also out performed junior residents during excision of the simulated lesion in LPNx, a finding that approached statistical significance (*p* = 0.07). There were no significant differences in performance time in any of the other steps.

## Discussion

By necessity, surgical education is moving away from the experience-based model of training to a more skills-oriented and procedure-based model (9–12). In this vein, we developed a comprehensive simulated training curriculum that culminates in live-tissue simulation of LRNx and LPNx. Given the increasing need to leverage clinical operative time for maximum learning, we hypothesized that our live-tissue model could be used to identify whether specific operative steps slow trainee progress more than others. In our models of LRNx and LPNx, senior residents completed the operations more quickly than did their junior counterparts. Notably, junior residents took significantly more time to complete renal hilar dissection than did seniors, while performing other steps at a similar pace. Now that this isolated performance differential in laparoscopic nephrectomy is known, specific efforts can be made to teach junior residents the skills required for effective and timely hilar dissection early in their training, thereby freeing up valuable time in the operative theater for the teaching of other important surgical skills.

Our study confirms what most urology faculty innately know – that the variability and delicacy of the vessels and the risk of serious complication makes the hilar dissection inherently challenging to teach clinically on a human patient. Indeed, Yap et al*.* showed that both residents and attending surgeons agree that resident participation during LRNx is significantly less in hilar dissection than in other aspects of the case (13). We feel our study supports two important and generalizable principles for surgical education: first, simulation enables educators to identify skills for targeted training and, second, live-tissue simulation allow junior residents to gain experience in operative maneuvers deemed too risky to be learned solely on human patients.

Though we are the first to identify a significant time-differential for an individual operative step, previous authors have examined variation in simulated surgical skills between senior and junior residents. Given that senior residents typically have substantially more laparoscopic and robotic operative experience, it is not surprising that seniority translates into shorter overall operative time in our live-tissue simulation models. The literature, in general, supports the notion that seniority shortens the time needed to gain proficiency in simulated surgical skills. Lee et al*.* simulated laparoscopic renal hilar vascular injury using an inanimate model, and found that senior residents scored higher on a global rating scale and had lower simulated blood loss (10). Similarly, in a team-based training exercise using a computerized human simulator, urologic resident training level was once again correlated with technical performance and blood loss (14). In contrast, Verdaasdonk found that previous laparoscopic experience in first and second year surgical residents did not correlate with the ability to master a simulated laparoscopic skillset and theorized instead that innate body-awareness, spatial reasoning skills, and motivation of the trainees influenced their ability to gain proficiency (15). Weizer et al*.* evaluated the time required for junior and senior residents to complete defined surgical steps in a series of hand-assisted laparoscopic donor nephrectomies supervised by a single surgeon (16). The authors found that after controlling for patient factors known to lengthen operative time (e.g. BMI and gender), junior residents still required significantly more time to mobilize the colon and the kidney and ureter when compared to senior residents. This difference also translated to a longer overall operative time. Though time to complete the hilar dissection was measured in this study of 70 consecutive patients, it is noteworthy that residents did not perform the dissection enough times to warrant analysis of their performance. As surgical educators, we must acknowledge the incongruity between the need to maintain quality and safety, and the need to allow trainees to gain critical experience, which inevitably will generate mistakes. Weizer's study highlights this struggle and emphasizes the value of surgical simulation for providing an environment in which complicated maneuvers can be practiced without putting human patients at risk.

As surgical education moves away from the experience-based model of training to a skills-based model, knowledge of the specific skills to target for maximal learning is critical. The development of high fidelity, lifelike simulator models provides safe, portable, inexpensive and readily-available training for a wide variety of surgical skills, while also lending insight into which skills are most in need of improvement. Indeed, both the learner and the instructor will benefit from this identification of strengths and weaknesses, information that can be used to make both the operative and simulated sessions even more educationally fruitful. While the live tissue model used herein is likely the highest-fidelity non-human model, it is also expensive and labor-intensive to deploy.

Miyata and their group also looked at VR laparoscopic radical nephrectomy simulation and concluded that the simulation allows for excellent skill assessment of trainees prior to entering the operating room (17). From the realm of gynecologic resident education, Jokinin also showcased the utility of virtual reality laparoscopic simulation with hysterectomy. Of note, while operative time did not reach statistical significance due to low number of study participants, residents who completed the intervention of the laparoscopic hysterectomy simulation performed their first live hysterectomy an average of 20 min faster (18). In another gynecologic example, Netter took training a step forward and identified that video-based self-assessment can improve surgical skills on virtual reality laparoscopic hysterectomy simulation (19). Both of these gynecologic studies highlight cost effective methods of improving surgical education outside the operating room. Taking cost considerations even further, Casas-Murillo utilized 3D-printed anatomical models of the cystic duct for training of laparoscopic cholecystectomy in a homemade laparoscopic simulator using silicon which showed promising results in terms of the native tissue-like composition of the material (20).

This study is not without limitations. Some may argue that speed should not be measured in the evaluation of surgical trainees, as the time required to complete an operative step, in and of itself, may not be an accurate gauge of competency. However, faster operative times were a surrogate for lower complication rates in several studies evaluating the learning curve of laparoscopic surgery (11, 21, 22). Indeed, we believe that in our high fidelity live-tissue model in a simulated operating room setting, speed provides a global measurement of operative efficiency, which incorporates not only technical skill, but also critical thinking and intraoperative decision-making. Therefore, we believe using speed as an endpoint does not jeopardize the quality of the resident's training experience or of our evaluation. Furthermore, evaluators (attending urologists) who timed the steps could not be blinded to the identities of the participating residents. While task-timing should be reasonably free from subjectivity, we cannot rule out the possibility that bias may have entered our results. Finally, though extensive efforts were made to standardize the model, live-tissue simulation will never be 100% reproducible across operative experiences, as the unique anatomy and physiology of each animal model will influence the residents' experience. Moreover, though a single resident was designated as the primary surgeon for each operation, a second resident acting as an assistant also participated in each case. The presence of an assistant was not formally evaluated and could have affected the simulation standardization. Additionally, comparing time for hilar control between junior and senior residents performing two different operations may have induced bias into the results. LRNx hilar dissection is typically more elaborate than LPNx which potentially added to the duration of time taken to achieve hilar control for junior residents. Finally, our study does lack the standardized assessment of resident surgical skill through critical points of the operation. Our study began prior to the advent of these objective forms of assessment and thus time was used as a surrogate marker for skill.

## Conclusions

In our live-tissue simulations of laparoscopic radical and partial nephrectomy, senior residents completed the operations substantially more quickly than junior residents. In an effort to improve the quality of urologic surgical skills instruction, we sought to ascertain whether the difference in operative speed between senior and junior residents was due to the juniors performing discrete operative steps more slowly vs. a globally-slower performance, without slowness being concentrated at a specific step. Ultimately, a marked performance differential was evident during renal hilar dissection, but not during other steps. Our study suggests that specific efforts should be focused on teaching junior residents the skills required for hilar dissection early in their training. This strategy may improve patient safety by shortening overall length of anesthetic and may also provide instructors greater flexibility to teach more complex surgical skills. Furthermore, this may indicate the live animal model served as a valuable intermediate step between virtual reality training and the actual operating room.

## Data Availability

The raw data supporting the conclusions of this article will be made available by the authors, without undue reservation.

## References

[1] Nauta RJ. Five uneasy peaces: perfect storm meets professional autonomy in surgical education. J Am Coll Surg. (2006) 202(6):953–66. 10.1016/j.jamcollsurg.2006.02.029 16735211

[2] Motola I Devine LA Chung HS Sullivan JE Issenberg SB. Simulation in healthcare education: a best evidence practical guide. AMEE guide No. 82. Med Teach. (2013) 35(10):e1511–30. 10.3109/0142159X.2013.818632 23941678

[3] Kaiser LR Mullen JL. Surgical education in the new millennium: the university perspective. Surg Clin North Am. (2004) 84(6):1425–39. vii. 10.1016/j.suc.2004.06.012 15501267

[4] Aggarwal R Balasundaram I Darzi A. Training opportunities and the role of virtual reality simulation in acquisition of basic laparoscopic skills. J Surg Res. (2008) 145(1):80–6. 10.1016/j.jss.2007.04.027 17936796

[5] Sewell C Morris D Blevins N Barbagli F Salisbury K. Quantifying risky behavior in surgical simulation. Stud Health Technol Inform. (2005) 111:451–7. PMID: 15718777

[6] Korndorffer JR Jr., Dunne JB Sierra R Stefanidis D Touchard CL Scott DJ. Simulator training for laparoscopic suturing using performance goals translates to the operating room. J Am Coll Surg. (2005) 201(1):23–9. 10.1016/j.jamcollsurg.2005.02.021 15978440

[7] Sturm LP Windsor JA Cosman PH Cregan P Hewett PJ Maddern GJ. A systematic review of skills transfer after surgical simulation training. Ann Surg. (2008) 248(2):166–79. 10.1097/SLA.0b013e318176bf24 18650625

[8] Heinrichs WL Lukoff B Youngblood P Dev P Shavelson R Hasson HM Criterion-based training with surgical simulators: proficiency of experienced surgeons. JSLS. (2007) 11(3):273–302. PMID: , PMCID: 17931510 PMC3015829

[9] Issenberg SB McGaghie WC Hart IR Mayer JW Felner JM Petrusa ER Simulation technology for health care professional skills training and assessment. JAMA. (1999) 282(9):861–6. 10.1001/jama.282.9.861 10478693

[10] Lee JY Mucksavage P McDougall EM. Simulating laparoscopic renal hilar vessel injuries: preliminary evaluation of a novel surgical training model for residents. J Endourol. (2012) 26(4):393–7. 10.1089/end.2011.0432 22192098

[11] Hung AJ Ng CK Patil MB Zehnder P Huang E Aron M Validation of a novel robotic-assisted partial nephrectomy surgical training model. BJU Int. (2012) 110(6):870–4. 10.1111/j.1464-410X.2012.10953.x 22313582

[12] Gurusamy KS Aggarwal R Palanivelu L Davidson BR. Virtual reality training for surgical trainees in laparoscopic surgery. Cochrane Database Syst Rev. (2009) (1):CD006575. 10.1002/14651858.CD006575.pub2 19160288

[13] Yap SA DeLair SM Tanaka ST Kurzrock EA. Current perceptions of resident training in laparoscopic nephrectomy. Urology. (2009) 73(5):1067–71. 10.1016/j.urology.2008.08.520 19394507

[14] Lee JY Mucksavage P Canales C McDougall EM Lin S. High fidelity simulation based team training in urology: a preliminary interdisciplinary study of technical and nontechnical skills in laparoscopic complications management. J Urol. (2012) 187(4):1385–91. 10.1016/j.juro.2011.11.106 22341287

[15] Verdaasdonk EG Dankelman J Lange JF Stassen LP. Incorporation of proficiency criteria for basic laparoscopic skills training: how does it work? Surg Endosc. (2008) 22(12):2609–15. 10.1007/s00464-008-9849-4 18389319

[16] Weizer AZ Ye Z Wolf JS Jr. Hollenbeck BK. Understanding potential intraoperative impediments for learning laparoscopic nephrectomy. J Endourol. (2008) 22(6):1339–44. 10.1089/end.2008.0439 18578662

[17] Miyata H Abe T Hotta K Higuchi M Osawa T Matsumoto R Validity assessment of the laparoscopic radical nephrectomy module of the LapVision virtual reality simulator. Surg Open Sci. (2020) 2(1):51–6. 10.1016/j.sopen.2019.08.003 33981981 PMC8083013

[18] Jokinen E Mikkola TS Harkki P. Simulator training and residents’ first laparoscopic hysterectomy: a randomized controlled trial. Surg Endosc. (2020) 34(11):4874–82. 10.1007/s00464-019-07270-3 31768724 PMC7572324

[19] Netter A Schmitt A Agostini A Crochet P. Video-based self-assessment enhances laparoscopic skills on a virtual reality simulator: a randomized controlled trial. Surg Endosc. (2021) 35(12):6679–86. 10.1007/s00464-020-08170-7 33241429

[20] Casas-Murillo C Zuniga-Ruiz A Lopez-Barron RE Sanchez-Uresti A Gogeascoechea-Hernandez A Munoz-Maldonado GE 3D-printed Anatomical models of the cystic duct and its variants, a low-cost solution for an in-house built simulator for laparoscopic surgery training. Surg Radiol Anat. (2021) 43(4):537–44. 10.1007/s00276-020-02631-3 33386458

[21] Kanno T Shichiri Y Oida T Kanamaru H Takao N Shimizu Y. Complications and the learning curve for a laparoscopic nephrectomy at a single institution. Int J Urol. (2006) 13(2):101–4. 10.1111/j.1442-2042.2006.01239.x 16563130

[22] Gill IS Kavoussi LR Clayman RV Ehrlich R Evans R Fuchs G Complications of laparoscopic nephrectomy in 185 patients: a multi-institutional review. J Urol. (1995) 154(2 Pt 1):479–83. 10.1097/00005392-199508000-00037 7609110

